# Death of Dr. Troost

**Published:** 1850-09

**Authors:** 


### THE WESTERN JOURNAL.

THIRD SERIES.—VOL. VI.—No. III.

LOUISVILLE, SEPTEMBER 1, 1850.

### DEATH OF DR. TROOST.

The intelligence of the death of Dr. Troost, the profound Naturalist, the able Professor of Chemistry and Geology in the University of Nashville, will be received with sorrow by the friends of science in every quarter of the globe. On the occasion of his death the following preamble and resolutions were adopted by the Trustees of the University:

"The Board having learned, with profound sorrow and regret, that Gerard Troost, M. D., Professor of Chemistry, Geology, and Mineralogy in the University, after a severe and protracted illness, departed this life on the 14th instant, at one o'clock, P. M., deem it due to the memory of the distinguished Professor and to his numerous surviving friends, to record their high appreciation of his eminent virtues and meritorious labors in the cause of science and philosophy.

"Born and liberally educated in Holland, he early manifested a zealous devotion to Natural History and Chemistry, and more especially to the then infant sciences of Geology and Mineralogy. With a view to the more successful prosecution of his favorite studies, he visited Paris; and, for several years, was a pupil of the celebrated Haüy. He removed to the United States about forty years ago; and, in due time, became an American citizen. His entire life was consecrated to Geology and the kindred sciences. With what ability and success, his published writings and his well-earned reputation at home and abroad, may eloquently testify.

"As a Professor in this University, during the last twenty-two years, and as the State Geologist of Tennessee for most of that period, he won the confidence and respect of the community, by invaluable services in both capacities, as well as by the unaffected modesty, kindness and uniform courtesy of his deportment towards all men. In the various stations and relations of life, public and private, he was without reproach and above suspicion. Beloved, trusted, honored, venerated, by all those most intimately connected or associated with him, he could not make an enemy—and he had none.

"His mineral and geological cabinets and other scientific collections—the fruit of half a century's patient research and judicious exchanges with the *savans* of both hemispheres—were, probably, the richest and largest ever possessed by any individual in our country.

"Without attempting, however, on this occasion, either a formal biography or eulogium, we cordially adopt the following resolutions:

"1. *Resolved*, That by the death of the venerable, learned and accomplished PROFESSOR TROOST, the University has been deprived of one of its most useful and exemplary instructors—the country of one of its most gifted citizens—and science of one of its most faithful votaries.

"2. *Resolved*, That we deeply sympathize with the family of the deceased, and tender to the mourning widow and children the assurance of our heartfelt sense of their irreparable bereavement.

"3. *Resolved*, That as a token of respect, we wear the usual badge of mourning for thirty days, and that we attend the funeral of the deceased.

"4. *Resolved*, That these proceedings be published, and that a copy thereof be communicated, by the Secretary, to Mrs. Troost."

Dr. Troost devoted his long life assiduously to the cultivation of science. He was a genuine naturalist, with all the enthusiasm, and all the purity and simplicity of character of the best specimens of his class. His love of natural history continued unabated to the last. Only a few weeks before the close of his life we saw him as we were returning from a visit to his favorite Perry county, the locality whence he had drawn so many of his choice fossils, and the interest with which he looked over our collection was as great as could have been felt by any ardent young naturalist with the prospect of a long life before him. He had just completed and sent off to the publishers his Memoir on the *Crinoidea*, a work upon which he has expended the labor of several years, and which will be looked for with interest by paleontologists in every quarter of the world. His collection of fossils is large and varied, and his cabinet of minerals is, perhaps, the richest to be found in any collection in our country. It is understood that his cabinet will be purchased by the University of Nashville, with the fame of which the name of Troost has been so long and honorably associated.

Dr. Troost had suffered for many months with diabetes, under which his vigorous frame was slowly wasting away, when, during the intensely hot weather in the month of August, he was seized with cholera morbus which soon carried him off. Dr. Troost is better known in Europe for his contributions to natural history than in his adopted country, most of his discoveries having been announced in the scientific journals of Paris. He has given to Tennessee a name in science wherever geology is cultivated in any part of the globe. It was fitting that on the occasion of the decease of such a man the newspapers of his city should be clothed in mourning—a compliment seldom paid to the mere man of science.

